# Patients with depression who self-refer for transcranial magnetic stimulation treatment: exploratory qualitative study

**DOI:** 10.1192/bjb.2018.49

**Published:** 2018-12

**Authors:** Martin Clarke, Sudheer Lankappa, Mark Burnett, Najat Khalifa, Charlotte Beer

**Affiliations:** 1 Nottinghamshire Healthcare NHS Foundation Trust; 2 University of Nottingham

**Keywords:** Transcranial magnetic stimulation, depression, self-referral

## Abstract

**Aims and method:**

As part of a larger clinical trial concerning the use of transcranial magnetic stimulation (TMS) for treatment-resistant depression, the current study aimed to examine referral emails to describe the clinical characteristics of people who self-refer and explore the reasons for self-referral for TMS treatment. We used content analysis to explore these characteristics and thematic analysis to explore the reasons for self-referral.

**Results:**

Of the 98 referrals, 57 (58%) were for women. Depressive disorder was the most commonly cited diagnosis, followed by bipolar affective disorder. Six themes emerged from the thematic analysis: treatment resistance, side-effects of other treatments, desperation for relief, proactively seeking information, long-term illness and illness getting worse.

**Clinical implications:**

TMS has recently been recommended in the UK for routine use in clinical practice. Therefore, the number of people who self-refer for TMS treatment is likely to increase as its availability increases.

**Declaration of interest:**

None.

## Depression

Approximately one-half of patients with depression do not achieve an adequate response to antidepressants.^1^ A review found that approximately one-third of patients with depression showed no response while some achieved only a partial response to treatment, as operationalised by a reduction in depressive symptoms.^2^

## Brain stimulation technique

Electroconvulsive therapy (ECT) is a well-established neuromodulation technique used to treat depression,^3^^,^^4^ but it is associated with side-effects such as memory deficits^5^ and risks associated with general anaesthesia. This has provided the impetus to develop other brain stimulation techniques, such as transcranial magnetic stimulation (TMS). TMS is a non-invasive brain stimulation technique that induces changes in cortical excitability using high-intensity magnetic pulses delivered through the scalp.^6^^,^^7^ It has been used to treat various neuropsychiatric conditions including depression. In the UK, repetitive TMS (rTMS) for depression has been recently recommended for use in routine clinical practice.^8^ In comparison with ECT, TMS treatment confers certain advantages, in terms of tolerability and ease of adminstration.^9^

## The TMS trial

The current study was conducted as part of a clinical trial (TDep TMS trial, Clinicaltrials.gov: NCT02016456), which sought to examine the effectiveness of repetitive high-frequency TMS versus intermittent theta burst TMS in reducing the symptoms of treatment-resistant depression. The trial was conducted under the auspices of the Nottingham Neuromodulation Unit. The lead trial investigator (S.L.) received emails from patients enquiring about the trial or requesting TMS treatment. Such enquiries were considered important given the generally poor rates of treatment-seeking and self-referral by people with depression.^10^

## The current study

The self-referral emails offered a novel opportunity to explore why people with depression self-referred for TMS. The current study aimed to explore the referral emails and describe the clinical characteristics of people who self-refer and the reasons for self-referral for TMS treatment.

## Method

### Design

This qualitative study used self-referral email correspondence to explore the characteristics of patients self-referring and the reasons for self-referral for TMS treatment. Emails had been sent by the self-referrer, their relative or their doctor, and requested TMS treatment for medical conditions (most often depression).

### Approvals

This study is part of a clinical trial which has both research ethics and Research and Development approval. Approval to review the content of the referral emails was granted by the Research and Innovation Department of the Nottinghamshire Healthcare National Health Service (NHS) Foundation Trust.

### Participants

The participants were people with a health condition, most commonly depression, seeking treatment or further information on TMS. The emails were often sent by the potential participant themselves; however, a few were sent by doctors or family members. These were also regarded as self-referrals because it was clear that they were written on the instruction of, or in collaboration with, the patient. All 98 self-referral emails received between the start of the trial in May 2014 and October 2015 were analysed.

### Analysis

Referral emails were redacted to obscure all identifiable patient information. They were analysed using content and thematic analysis. Details about participant characteristics were briefly stated in the emails or in some instances inferred from details such as name, and so content analysis was used to extract and analyse these data. Thematic analysis was used to explore reasons for self-referral, as this approach enables the researcher to explore themes both inductively from the data and deductively based on theory and research.^11^ Analysis followed Braun and Clarke's six phases of thematic analysis.^11^ Emails were read and re-read (by M.B.) and, after familiarisation with the data, codes were generated by coding interesting and shared features in a systematic way across all the emails. Codes were sorted into potential themes and the coded extracts were collated into these themes. Analysis proceeded iteratively and was refined in collaboration with another qualitative researcher (C.B.). Themes were defined and coded, following accepted guidelines.^12^ Interrater reliability of coding was also assessed; 89% interrater reliability was achieved (scores >70% are considered acceptable).^12^

## Results

### Participant characteristics

Of the 98 referral emails analysed, in 90 (91.8%), it was clear whether the potential participant or someone else had written the email. Of these, the majority (78.9%, *n* = 71) were written by an individual who was applying to take part in the TMS treatment, with the others written by doctors (11.1%, *n* = 10) or family members on behalf of the individual (10.0%, *n* = 9). Gender was explicitly stated, or could be inferred from the name, for 83 referrals (84.7%). Of these, there were 48 women (57.8%) and 35 (42.2%) men. Age was provided for 31 referrals (31.6%). The mean age was 44 years (range early 20s to mid-70s). The length of illness was given for 33 referrals (33.7%). Where stated, the mean length of illness was 17 years (range 4 months to more than 40 years). The primary diagnosis was specified for 63 referrals (64.3%). Depression was the most commonly cited diagnosis (88.9%, *n* = 56), followed by bipolar affective disorder (6.3%, *n* = 4) and schizophrenia (4.8%, *n* = 3). Twenty referrals (20.4%) mentioned comorbidities, the most common being anxiety (50.0%, *n* = 10).

### Reasons for self-referral

Thematic analysis of the data revealed six themes that explained the reasons for self-referral for TMS treatment. The self-referral emails varied widely in the depth of detail provided, and themes were expressed in very diverse ways. Given that there were 98 emails, the prevalence of themes was reported (Table 1). This is important since a powerful and memorably described theme might assume disproportionate importance.

**Table 1:** List of themes

Theme	Number of participants identifying with theme
1. Current treatment not working	39 (39.8%)
2. Proactively seeking information about treatment for depression	29 (29.6%)
3. Suffering from chronic or long-term depression	25 (25.5%)
4. Desperate for relief from depression	13 (13.3%)
5. Motivated to seek alternative treatment owing to side-effects of current or previous treatment	12 (12.2%)
6. Getting worse in spite of current treatment regime	6 (6.1%)

### Current treatment not working

This theme was coded in 40% of the emails. The most commonly cited reason for self-referral for the TMS trial was lack of, or only limited response to, treatment despite undergoing various treatment modalities. For example, one email described constant relapsing even after many different treatments.

I have tried various medications, CBT and Mindfulness but I relapse again and again (W7, Female).

Another email described how treatment, including ECT, had never worked, even partially or for a short period of time.

I have tried at least 20 different types of anti-depressant tablets … none of which have worked. I have also has [sic] 2 courses of ECT and several years of CBT (both group and individual) all with no effect (M15, Male).

This theme reflects the definition of treatment resistance used in the wider study, namely the ‘failure to improve or only partially improve after trying two or more antidepressants or two or more psychotherapies/ECT’. Indeed one writer explicitly stated that her mother's depression was treatment resistant.

… has suffered with what is proving to be treatment resistant depression for over 12 months (W61, Female).

### Suffering from chronic or long-term depression

Another important motivator for self-referral was experiencing chronic or long-term depression, with this theme coded in a quarter of the emails. For example, one writer stated that he had suffered with depression for more than 20 years.

I have been suffering with depression for over 20 years (M30, Male).

### ‘Desperate’ for relief from depression

For some participants, their self-referral was prompted by their desperation for relief from depression (13%). One writer powerfully described how he was not even really ‘living’ and was desperate for this to happen.

I am desperate of finding a way to start living and enjoining [sic] life again (M3, Male).

Another writer described their increasing state of despair.

As I feel that age and time are against me, and I feel that I am slipping further and further down the well of total despair (M15, Male).

These calls for help are all the more powerful given that they were sent to a clinician with whom the patient had no therapeutic relationship. Such desperation is clear in the following plea.

Sir, I have no idea where to turn next, please offer me some help and hope before my marriage is stretched beyond it's [sic] tolerance – before it is too late (M7, Male).

For some, this desperation was caused by depression affecting their ability to function normally. This impaired functionality often affected the participant's ability to work. It also manifested in other ways such as problems with social functioning and lack of motivation. One email described how the writer had previously had a good career, but depression had significantly affected this and resulted in him losing his job.

Although I had a good professional career, 2 postgraduate degrees, my personal, social and working life is seriously limited by depression (I lost my job as well) (M3, Male).

Another email described how his social life had been affected so badly that he had cut himself off from those around him.

Have not been able to work since and have now become a recluse cutting myself off from family and friends (M20, Male).

Another writer talked about how upsetting they found their lack of functionality.

I am finding it very tedious and upsetting that I am unable to do the things I would [want] to do and need to do at times (W46, Female).

### Motivated to seek alternative treatment by side-effects of current or previous treatment

Self-referral was sometimes prompted by a desire to seek an alternative treatment to avoid side-effects. TMS is a treatment with few side-effects; these include headache, nausea, tiredness, syncope and, very rarely, epileptic seizures. As such it was attractive to people who had previously experienced negative side-effects with other treatments. Participants were clearly aware of this and referred in their emails to the intolerable side-effects they had previously experienced.

I was on medication for several years but after coming off I have found it impossible to get back to a medication without intolerable side effects (W1, Female).

Some participants had received ECT treatment and also had problems with the severe side-effects associated with it. TMS is recognised has having fewer severe side-effects, which is probably why the opportunity to receive TMS was being explored. For example, one email described a patient who had previously tried ECT but could not tolerate it so was looking for a similar treatment but with less severe side-effects.

I attempted ECT with her which she could not tolerate (W11, Female).

### Getting worse in spite of current treatment regime

A small number of people requesting TMS mentioned that their symptoms were worsening despite treatment and that this is what prompted their request to participate in the trial (6%). While this theme appeared in only six emails, it powerfully describes the effect of progressive deterioration on people's lives, as in the account below of a patient's relative.

She is now living a twilight existence and progressively deteriorating (W61, Female).

### Proactively seeking information about treatment for depression

An interesting inductive theme that emerged from the data was that for some individuals, the self-referral was motivated by a desire to try newer, unconventional, treatments for their illness (29.6%). Proactive searching of the internet or health-related articles in papers and magazines for novel treatments was how these individuals had found out about the TMS trial.

Many of the patients had significant knowledge about TMS, having previously researched TMS treatment. This theme illustrates how patients with depression wish to be actively involved in exploring treatment choices which are not offered by their general practitioner or psychiatrist. For example, one patient described how they had been researching TMS compared to other treatments they had been offered and had found that it could be better.

I have read and looked at articles regarding TMS and they look promising to cure depression with much better success rate than medications alone or medication and counselling (W7, Female).

Another patient had been offered ECT but, having researched TMS, decided that TMS was superior to ECT.

I have researched ECT and found that TMS seems to [be] much superior but still evolving as a treatment for depression (M20, Male).

## Discussion

Depression is one of the most commonly cited causes of morbidity worldwide,^13^ with a lifetime prevalence of approximately 8–12%.^14^^,^^15^ Depression can result in suicide,^16^ which accounts for 1.4% of all deaths worldwide.^17^ To our knowledge, this is the first study to describe the characteristics of people with depression self-referring and the reasons for self-referral for TMS treatment, albeit in the context of a trial.

Content analysis of the emails revealed that self-referrers were a heterogeneous group. There was a broad age range, indicating that TMS has a broad appeal across all age groups. Participants had generally experienced their illness for several years. However, the average illness length may be confounded if those who included their length of illness were those who had experienced it for longer. For example, participants may have included their long length of illness to emphasise its severity, perhaps in the belief that it would increase their chance of being accepted onto the trial.

More women than men self-referred for TMS treatment. This is in line with research that has shown that women are more likely than men to seek help for mental disorders,^18^^,^^19^ and that depression is more prevalent in women than men.^20^

Thematic analysis of the emails revealed a number of factors prompting self-referral. The themes offer some important insights into what motivates people with depression to enquire about TMS treatment. The most common theme that emerged was ‘current treatment not working’. Other related themes included ‘motivated to seek alternative treatment owing to side-effects of current or previous treatment’, ‘suffering from chronic or long-term depression’ and ‘getting worse in spite of current treatment regime’. The side-effects of treatments with antidepressants have been well documented^21^^,^^22^ and so this was not an unexpected theme. The chronicity and possible long-term nature of depression are also well documented^23^ and perhaps unsurprisingly this was mentioned in one-quarter of self-referrals. Self-referrers also reported that their symptoms were ‘getting worse in spite of a current treatment regime’. Although this theme was the least common, revealed in only six emails, it was powerfully described and resonates with the literature on why people seek help,^24^ and so should not be ignored.

A related and powerful theme was ‘desperate for relief from depression’. Participants movingly described their desperation for relief from depression. The lack of treatment options, and not just symptom severity, may contribute to feelings of desperation.^25^ Evidence of such themes need not exclude participants from trials; as Swift^26^ commented, desperation affects voluntariness rather than capacity to enter into a trial, and this is related to whether acceptable alternative treatments are available. Moreover, Dunn and colleagues argued that including desperate patients in clinical trials is ethical.^25^

The themes described above go some way towards explaining why, despite their depression, participants were actively seeking information about treatment. The unexpected and inductive theme ‘proactively seeking information about treatment for depression’ revealed how participants had found information about the TMS trial while researching alternative treatments.

To date, no published studies have examined why patients with depression self-refer for TMS. However, gaining access to additional services, such as otherwise unavailable interventions, has been identified as a key facilitator for recruiting people with depression into clinical trials.^27^ Although TMS was not discussed, the authors noted a preference for interventions that did not involve medication.^27^ This has important implications for recruitment and resonates with Locock and Smith's study, which found personal benefit to be a primary motivation for volunteering in a research study, more so than altruistic considerations.^28^ Their study explored the reasons people volunteered to participate in clinical trials across different (mainly physical) conditions, and found that such personal benefits included access to new treatment, access to better information and receiving care from expert specialised teams.^28^

### Limitations

The main limitation of this study was the availability of data. Analyses were constrained by the information available in the initial referral email. Emails tend to be short and contain only information the writer wishes to share. Accordingly, there was no opportunity to clarify information with the participants, and relevant information may have been omitted in the referral email. For example, there were missing data for some of the participant characteristics. Furthermore, the prospective provision of TMS was linked to a clinical trial where participants received an intervention 4 days a week for 4 weeks. Potential self-referrers may have been restricted by this costly and time-consuming commitment, especially for those who lived further afield. However, while these constraints may have had an influence on who ultimately participated in the trial, the email writers were enquiring about, rather than enrolling in, the study. Therefore, the participant demographics may still be credible. Future research should include interviews with participants to explore their reasons for referral to TMS, to see whether similar reasons are identified which support our findings. There was also no opportunity for participants to provide feedback on the findings. It would also be informative to explore whether participants’ reasons for referral were related to their response to TMS. However, given that TMS is a relatively new technique and is not yet widely used in the NHS, we are not aware of any previous research on the characteristics of those who request the treatment or their reasons for referral to it, particularly in the UK.

### Summary

In conclusion, the 98 people who self-referred for TMS were a heterogeneous group, although the majority were female (57.8%). Thematic analysis of the self-referral emails revealed that participants were motivated by a desire for an effective alternative treatment for their treatment-resistant depression. These findings have implications for how participants for future TMS trials could be targeted; they also suggest an increased demand for TMS as it becomes more widely known. Given the updated guidance recommending rTMS for depression in routine clinical practice in the UK^8^, it is likely that more treatment centres will be developed to facilitate this.^29^ Prior to this, rTMS was only administered in research settings as National Institute for Health and Care Excellence guidelines stated that although TMS was judged to be safe, there was uncertainty about the clinical efficacy.^6^^,^^30^

## About the authors

**Martin Clarke** is a Research Fellow at Nottinghamshire Healthcare NHS Foundation Trust and Honorary Assistant Professor at the School of Medicine, University of Nottingham, UK; **Sudheer Lankappa** is a Consultant Psychiatrist at Nottinghamshire Healthcare NHS Foundation Trust and Honorary Clinical Associate Professor at the School of Medicine, University of Nottingham, UK; **Najat Khalifa** is a Consultant Forensic Psychiatrist at Nottinghamshire Healthcare NHS Foundation Trust and an Associate Professor at the School of Medicine, University of Nottingham, UK; **Mark Burnett** is a Medical Student at the School of Medicine, University of Nottingham, UK; **Charlotte Beer** is an Assistant Professor at the School of Medicine, University of Nottingham, UK.
